# Supplementary material for: Stress distribution on different bar materials in implant-retained palatal obturator
Source: PLoS One. 2020 Oct 30;15(10):e0241589. doi: 10.1371/journal.pone.0241589 (PMC7598468; doi:10.1371/journal.pone.0241589)
Supplement: Attachment — Submitted filename: Answer to reviewers.docx [file pone.0241589.s001.docx]

Please use the space provided to explain your answers to the questions above. You may also include additional comments for the author, including concerns about dual publication, research ethics, or publication ethics. (Please upload your review as an attachment if it exceeds 20,000 characters)

Reviewer #1: This paper presents the results of the numerical investigations on the mechanical response of implant-retained obturator prostheses with bar-clip attachment and milled bars. The research topic is quite important and well suited in the journal scope. The results obtained are well interpreted. Moreover, structure of this manuscript and graphic quality of figures are acceptable.
There are some more comments from this reviewer that can help the Authors to improve the quality of the manuscript. Minor revisions are suggested. The detailed comments in the order of appearance in the text are summarized as follows.

The novelty of this manuscript has not been clearly specified. I found many similar numerical analyses in the literature. The elastic properties of materials are assumed basen on the literature. External hexagon implants (10 × 4.1 mm), previously modeled [25] were selected. An anteroposterior structure was used crossing the hemimaxillectomy defects similar to [11]. report. The value of simulated loads is based also on the literature. etc., etc., So, what is the novelty of this manuscript???

R: First and foremost, we would like to thank the referee for the constructive criticisms, which have contributed to improve the content and the style of the manuscript. The reported parameters are used to ensure a well-controlled modelling process and it is common in studies like that. However, the finite element analysis of a hemimaxillectomy defect with this prosthesis design and different materials were never reported before in literature. Also, it is important to note that the implant model was previously reported but not in this kind of oral rehabilitation as you can see in the reference 25. In the same way, the reference 11 that was used as hemimaxillectomy defect reference is a case series, which have not evaluated the mechanical response.

2. Definition of the materials is too general. In the Abstract the authors say that "PEEK, titanium and Co-Cr alloys" were considered. The grades of titanium and Co-Cr alloys and/or chemical composition must be clearly specified.
R: This information has been inserted in the text.

3. Line 30: "PEEK". Please define all abbreviations the first time they appear in the abstract, and the main text.

R: The PEEK definition has been inserted in the text.

4. The method of determination of optimal mesh size is very mysterious. Line 122: "A convergence test of 10% determined [...]". 10% of what?

R: 10% of difference in results between each calculation. This information has been inserted in the methods section.

5. Conclusions are to general. The conclusions must be drawn appropriately based on the data presented.

R: The conclusion section has been improved.

Reviewer #2: Review comment
This manuscript entitled “Stress distribution on different bar materials in implant-retained palatal obturator” primarily aimed to evaluate the mechanical response of implant-retained obturator prostheses with bar-clip attachment and milled bars in three different materials: PEEK, titanium and cobalt-chromium alloys in different load incidences. The authors bring an interesting study. However, all the issues that listed below must be revised thoroughly before this manuscript being accepted for publication. I give a major revision for this manuscript.
Specific comments
Abstract
1. Abbreviations that exist in manuscript should be explained when they first appear, such as “PEEK” (Line 30). Please add the official explanation.

R: The abbreviation has been explained in the text.

2. The detailed modeling method can be presented in the method session, the authors should give more information regarding the results, conclusion and application (if possible) of this study in this part.’

R: Thank you. The abstract section has been improved which more emphasis in the results and shorten modeling process.

Introduction
1. “PEEK has shown some advantages such as…, which are usual materials in restorative dentistry” (Line 65-68), some references should be added.

R: References were inserted in this statement.

2. The novelty of this study should be further highlighted in the introduction part.

R: An improvement in the study’s novelty has been performed in the line 73.

Methods
1. It is suggested that a flow chart for the pre-processing is needed in order to help readers better understand the methods.

R: A new figure 1 was inserted with a flow chart for the pre-processing step.

2. Did this study apply any statistical methods to compare the differences between materials? If did, please specify it, and if not, please explain how the significant results came out without any statistical analysis.

R: As the finite element method already provide a calculated results, there is no need to apply a posterior statistic test. In order to predict difference between the groups, the stress maps can be compared qualitatively and the stress peaks (Table 2) assumed as “significant different”, instead statically signiant difference, if this difference was higher than 10% (The mesh convergence, line 129. This result evaluation and interpretation is well defined and accepted in literature, as you observe in studies with similar methodology.

Results
1. The results session should be rewritten based on the statistical analysis.

R: Thank you for your suggestion, however it is not applicable for the present study.

Discussion
1. “FEA” (Line 271), although I know the meaning of this abbreviation, the official explanations should be added.

R: Thank you for your observation. The abbreviation has been explained in the text.

2. “Computer-assisted implant planning has become …the most suitable mechanical results” (Line 282-285), this paragraph should be moved to the introduction part, as it seems to be the novelty of this study.

R:This paragraph has been moved to the introduction section.

Conclusion
1. Are there any implications based on the results of this study? Please specify.

R: The conclusion has been modified to: “For this treatment modality, regardless the loading region, PEEK can be suggested as framework material to reduce the bone strain around the implants and the stress concentration in the bar structure. However, the use of PEEK increases the risk of prosthetic screws loosening and even fracture in comparison with metallic alloys”.

While written English is reasonably good, but please do check the language and grammar mistakes throughout the whole article to improve clarity.

R: The language has been reviewed.
