# Supplementary material for: Stress distribution on different bar materials in implant-retained palatal obturator
Source: PLoS One. 2020 Oct 30;15(10):e0241589. doi: 10.1371/journal.pone.0241589 (PMC7598468; doi:10.1371/journal.pone.0241589)
Supplement: Attachment — Submitted filename: Answer to reviewers.docx [file pone.0241589.s002.docx]

Please use the space provided to explain your answers to the questions above. You may also include additional comments for the author, including concerns about dual publication, research ethics, or publication ethics. (Please upload your review as an attachment if it exceeds 20,000 characters)

Reviewer #2: The authors have addressed the comments carefully. However, there are still some questions that must be revised before this manuscript being accepted for publication. I give a minor revision for this manuscript.

Specific comments

1. Please merge the fourth and fifth paragraphs of the Introduction session further.

R: These paragraphs were merged as suggested.

2. The purpose of this study was to evaluate the mechanical response of implant-retained obturator prostheses with bar-clip attachment and milled bars in three different materials under two load incidences, however, from the conclusion (Abstract and Conclusions part), it seems that the authors only indicated some advantages and disadvantages of PEEK material, what about the other two materials? Any differences? Please specify.

R: Yes. As we can observe in the results section. Since PEEK is the novelty, our conclusion was based on its mechanical response. However, the conclusion section has been improved.

3. Abbreviations that exist in main content of this manuscript only need to be explained when they first appear, Line 251 polyetheretherketone (PEEK), Line 292 finite element analysis (FEA), please modify.

R: The abbreviation explaining has been already presented in the first apparition of PEEK, in line 65. The first apparition of FEA was in line 79 and the abbreviation explaining has been already presented too.
